# Tumor Growth Limiting Effects of Piceatannol

**DOI:** 10.1155/2013/514349

**Published:** 2013-03-26

**Authors:** Shailendra Kapoor

**Affiliations:** 74 Crossing Place, Mechanicsville, VA 23221, USA

Kita et al. have provided interesting data in their recent article [1]. Piceatannol may attenuate tumor growth in a number of systemic malignancies besides hepatocellular carcinomas. 

Similar effects are seen in prostatic malignancies. It mediates this role by augmenting tumoral apoptosis. There is increased cytochrome C release from the mitochondria. Piceatannol also causes downregulation of mTOR [2]. Piceatannol also has an inhibitory effect on IL-6/STAT3 signaling that further accentuates intratumoral apoptosis [3]. Besides these effects, piceatannol also decreases the release of VEGF. A simultaneous decrease in Akt and eIF-4E-BP1 accompanies the above changes. As a result, there is increased G1 phase arrest. Bik and Bok expression is also enhanced. “Urokinase-type plasminogen activator” secretion is also decreased [4]. The net result is that that tumor invasiveness is markedly attenuated. Tumor metastasis is markedly inhibited at the same time. Also, there is downregulation of PARP expression within the tumor cells. The levels of cleaved caspase-7 are augmented at the same time. At the same time Bcl-xL levels are downregulated while Bax levels are upregulated [5]. CDK4 and CDK2 activity is also attenuated. 

Similarly, piceatannol attenuates tumor growth in breast carcinomas. It mediates this role by significantly inhibiting MMP-9. Similar inhibition of the NF-*κ*B pathway accompanies the above changes [6]. I*κ*-B*α* phosphorylation is also attenuated resulting in decreased nuclear translocation of NF-*κ*B. It also has an inhibitory effect on the PI3K/AKT pathway. Similar effects are seen in colorectal malignancies. Cyclin B1 expression is decreased secondary to piceatannol administration. Accumulation of cancerous cells in the S phase is accentuated. p27 (Kip1) levels are also downregulated [7]. Cyclin D1 expression is also decreased. 

The above examples clearly indicate the significant tumor attenuating effects of piceatannol. 
